# What are the anticipated benefits, risks, barriers and facilitators to implementing person-centred outcome measures into routine care for children and young people with life-limiting and life-threatening conditions? A qualitative interview study with key stakeholders

**DOI:** 10.1177/02692163241234797

**Published:** 2024-03-13

**Authors:** Hannah May Scott, Lucy Coombes, Debbie Braybrook, Daney Harðardóttir, Anna Roach, Katherine Bristowe, Myra Bluebond-Langner, Lorna K Fraser, Julia Downing, Bobbie Farsides, Fliss EM Murtagh, Clare Ellis-Smith, Richard Harding

**Affiliations:** 1 King’s College London, Florence Nightingale Faculty of Nursing Midwifery and Palliative Care, Cicely Saunders Institute, London, UK; 2 Royal Marsden NHS Foundation Trust, London, UK; 3 University College London, London, UK; 4 University College London, Louis Dundas Centre for Children’s Palliative Care, London, UK; 5 Rutgers University, Camden, NJ, USA; 6 International Children’s Palliative Care Network, Kampala, Uganda; 7 Brighton and Sussex Medical School, University of Sussex, Brighton, UK; 8 Wolfson Palliative Care Research Centre, Hull York Medical School, University of Hull, Hull, UK

**Keywords:** Paediatrics, palliative care, children, implementation science, patient reported outcome measures, patient-centred outcomes research

## Abstract

**Background:**

There is a growing evidence-base underpinning implementation of person-centred outcome measures into adult palliative care. However evidence on how best to achieve this with children facing life-threatening and life-limiting conditions is limited.

**Aim:**

To identify the anticipated benefits, risks, barriers and facilitators to implementing person-centred outcome measures for children with life-limiting and life-threatening conditions.

**Design:**

Cross-sectional qualitative semi-structured interview study with key stakeholders analysed using Framework analysis informed by the adapted-Consolidated Framework for Implementation Research.

**Setting/participants:**

A total of *n* = 26 children with life-limiting or life-threatening conditions, *n* = 40 parents/carers, *n* = 13 siblings and *n* = 15 health and social care professionals recruited from six hospitals and three children’s hospices and *n* = 12 Commissioners of health services.

**Results:**

All participants were supportive of future implementation of person-centred outcome measures into care. Anticipated benefits included: better understanding of patient and family priorities, improved communication and collaborative working between professionals and families and standardisation in data collection and reporting. Anticipated risks included increased workload for staff and measures not being used as intended. Implementation barriers included: acceptability and usability of outcome measures by children; burden and capacity of parents/carers regarding completion; privacy concerns; and language barriers. Implementation facilitators included designing measures using language that is meaningful to children and families, ensuring potential benefits of person-centred outcome measures are communicated to encourage ‘buy-in’ and administering measures with known and trusted professional.

**Conclusions:**

Implementation of person-centred outcome measures offer potential benefits for children with life-limiting and life-threatening conditions. Eight recommendations are made to maximise benefits and minimise risks in implementation.

**What is already known on this topic?**

- Person-centred outcome measures have been shown to improve the quality of care and patient outcomes in adult palliative care when successfully implemented into routine care.
- Several factors influence implementation in adult services, but they have not been identified in care for children with life-limiting and life-threatening conditions.
- The views of stakeholders are key to successful development, implementation and use of outcome measures in practice.

**What this paper adds?**

- Perceived benefits of using person-centred outcome measures include enhanced understanding of what matters to patients and families, improved communication and collaborative working and standardised data collection and reporting; perceived risks include negative impacts on care and measures not being used as intended.
- Potential barriers to implementation include acceptability and usability of the measure for children, burden and capacity of patients and families to complete the measure, privacy concerns, protecting family members and language barriers; potential facilitators include explaining the benefits of person-centred outcome measures and securing ‘buy-in’, measures being implemented by known and trusted health and social care staff and the language in the measure being meaningful to children and families.
- Eight recommendations are presented to minimise risks and support successful implementation of child and family-centred outcome measures for children with life-limiting and life-threatening conditions.

**Implications for practice, theory or policy**

- The benefits of person-centred outcome measures for care should be explained to children, families and professionals to facilitate buy in and successful implementation.
- Implementation strategies should be designed collaboratively with professionals to ensure implementation of person-centred outcome measures is feasible within current practice and does not impact negatively on care.
- Professionals introducing and administering the measure should be known and trusted by the child and family, and should discuss usage preference and information sharing to address any privacy concerns.

## Background

Person-Centred Outcome Measures (PCOMs) are standardised questionnaires that assess the effect of a health condition or treatment on the patient, and/or their family.^1–3^ They are usually self-completed (as with Patient Reported Outcome Measures (PROM)), or when the patient is unable, proxy-completed by a caregiver.^1–3^ Using PCOMs can empower patients and families to raise concerns with clinicians, and support conversations and decision-making through a shared language.^2,4–6^ These processes improve quality of care and patient outcomes.^7,8^

An estimated 21 million children and young people (hereafter ‘children’) worldwide with life-limiting and life-threatening conditions (hereafter ‘life-limiting’) could benefit from palliative care each year.^9^ Whilst there is a growing body of evidence on the use and implementation of PCOMs in adult palliative care,^1–3,10,11^ evidence to underpin their use and implementation for children with life-limiting conditions is more limited,^2,12–14^ particularly outside of paediatric oncology.^15–22^ Whilst there are several PCOMs (including both generic tools and condition specific measures) that have been developed, validated and implemented across paediatrics,^23–25^ available generic tools (e.g. Paediatric Quality of Life Inventory^14,26–32^) do not reflect the concerns of all children with life-limiting conditions, and condition specific measures (e.g. Memorial Symptom Assessment Scale^33,34^) are only relevant for their specific population, and therefore not suitable for use across all children with life-limiting conditions.^35^ Following development and initial validation of the Children’s Palliative care Outcome Scale: African version,^36–40^ development of a validated measure that can be used by all children with any life-limiting conditions outside of Africa, has been highlighted as a priority for clinical care and research.^41–46^

The CPOS:UK (Children’s Palliative care Outcome Scale: UK version) study aims to develop, validate and implement a novel PCOM for all children with any life-limiting condition in the UK. Five initial versions of the C-POS:UK have been developed to reflect variation in age/developmental stages of the target population and allow for proxy reporting if required^47–50^ following the Consensus-based Standards for the selection of health Measurement Instruments (COSMIN)^52,53^ and Rothrock guidance.^54^ However, future implementation to be successful, implementation strategies must be informed by the views and preferences of key stakeholders: children with life-limiting conditions, their family members and professionals involved in their care.

## Methods

### Research questions

RQ1: What are the anticipated benefits and risks of using a PCOM in the care of children with life-limiting conditions?
RQ2: What are the potential barriers and facilitators to implementing a PCOM in in the care of children with life-limiting conditions?

### Design

This cross-sectional qualitative interview study^47^ is reported in accordance with the consolidated criteria for reporting qualitative studies (COREQ).^55^ It sits within a sequential mixed-methods study to develop,^35,47–49,56–59^ validate^51^ and implement^60–62^ a novel PCOM for children with any life-limiting illness. Qualitative interviews were conducted during the development phase with the aims of identifying priority items to include in the measure,^47^ preferences for design and administration modes,^49^ and potential benefits and challenges of implementing a new PCOM into routine care. The data related to implementation collected during the development phase are reported here and will inform the implementation phase.

PCOMs are complex interventions (particularly in their usage to drive assessment, care and evaluation). Consistent with the Medical Research Council’s framework for developing and evaluating complex interventions, all key stakeholder groups views were sought to inform the design of the measure and the development of an implementation strategy.^1–3,10,11^ This work took a child- and family-centred approach which recognises the role of parents and the family in care planning and delivery while ensuring that the child’s voice and perspective is heard and fully considered.^63–65^

### Population

#### Inclusion criteria

Children: aged 5–17 years with any life-limiting condition.^66^

Parents/carers: parent or carer of a child aged <18 years with any life-limiting condition.^66^

Siblings: aged 5–17 years and sibling to a child <18 years with any life-limiting condition.^66^

Professionals: any professional with >6 months experience caring for children <18 with any life-limiting condition.^66^

Commissioners (responsible for planning, prioritising, purchasing and monitoring of services): responsible for commissioning UK paediatric palliative care services.

#### Exclusion criteria

Children: unable to communicate any views or wishes; speak a language not supported by NHS translation services; currently enrolled in another study; deemed clinically unable to give consent/assent.

Parents/carers and siblings: deemed clinically unable to give consent/assent or speak a language not supported by NHS translation services.

### Setting

Six hospitals (five with specialist consultant-led paediatric palliative care teams) and three children’s hospices (one with a specialist consultant-led paediatric palliative care team, two with nurse led services) across England and Northern Ireland.

### Sampling

Children and their families were purposively sampled by age and condition (i.e. cancer and non-cancer^38^). Professionals were purposely sampled to ensure a range of different professions were represented and commissioners were purposely sampled based on geographical location. The concept of information power or pragmatic saturation^67^ was used to determine the required sample size to address all of the aims of the original study.^47,49,60,61^ Due to the range of aims being met and the heterogeneity of the population, the dataset required diversity and depth.

### Recruitment

Potential child, parent/carer and sibling participants were identified by the local clinical team. Professionals were also recruited from these sites, identified by their service manager. Commissioners were recruited through recommendations from professionals and a national children’s palliative care non-governmental organisation.

### Data collection

Semi-structured interviews were conducted between April 2019 and September 2020 by LC (experienced children’s palliative care nurse, new to qualitative research), AR (new to qualitative research) and DB (experienced qualitative researcher).

Topic guides were developed collaboratively with the study steering group which includes clinicians of various professions (including doctors, nurses, social workers and other allied health professionals) working with children with life-limiting conditions, academics (including clinical academics) and bereaved parent public and patient involvement members. Further information on and examples of the topic guides are included in the Supplemental Files.

Interviewers received training and supervision on conducting interviews with children, including ‘draw and talk’ and play methods from an educational psychologist and play therapist. Participants were offered choice of location for face-to-face interviews of their clinical setting or their home (during the COVID-19 lockdown, interviews were only conducted by telephone^57^). Interviews were audio-recorded, transcribed verbatim and pseudonymised.

### Data analysis

Analysis followed the seven-step Framework method^68^; transcription, familiarisation, coding, developing an analytical framework/codebook, applying the framework, charting into the framework matrix and interpretation. Full transcripts were coded^68,69^ by LC, DB, AR, HS (experienced qualitative researcher) and DH (new to qualitative research) using NVivo 12 Software. About 20% of transcripts were independently coded by two researchers for consistency and rigour.^70^ Regular team (LC, DB, AR, HS and DH) meetings were held to discuss emerging codes/themes to develop and revise a codebook.^71^ RH, KB and CES were consulted to resolve discrepancies. The codebook was developed through 18 revisions, applied to all transcripts and data charted into a matrix generated by HS using NVivo12, which supports comparisons across and between groups.

A second phase of deductive analysis and interpretation was performed by HS through mapping the coded, charted data in the matrix to the domains of the adapted-Consolidated Framework for Implementation Research (CFIR).^72,73^ This supported the identification of the anticipated benefits, risks, barriers and facilitators relating to the implementation of PCOMs into paediatric palliative care. The CFIR comprises five domains of implementation: intervention characteristics (aspects of PCOMs that might affect implementation success), outer setting (external influences on implementation of a PCOM), inner setting (characteristics of the healthcare setting implementing a PCOM), characteristics of individuals (individual beliefs, knowledge and attitudes of stakeholders towards a new PCOM and its implementation) and process (stages of the implementation process that can impact implementation success). The adapted-CFIR includes a sixth domain, ‘patient needs and resources’^72,73^ (the extent to which patient’s needs, as well as barriers and facilitators to meet those needs, are known and prioritised by the healthcare setting) integrating person-centredness into the implementation of complex healthcare interventions.^73^ Analysis was regularly reviewed by the study steering group.

### Ethical approvals and consent

Ethical approval was granted by the Bloomsbury research ethics committee and the Health Research Authority (HRA:19/LO/0033). Participants ⩾16 years old provided written informed consent. Those with parental responsibility provided written informed consent for participants <16 years. Those <16 years were given the opportunity to provide written assent.

## Results

### Sample characteristics

We conducted 104 interviews with 106 participants (2 parents and 2 siblings were interviewed together): 26 children, 40 parent/carers, 13 siblings, 15 professionals and 12 commissioners. Demographic characteristics are reported in Table 1.

**Table 1.** Participant characteristics.

Children (*n* = 26)	*N* or mean (range)	Parent/carers (*n* = 40)	*N* or mean (range)	Siblings (*n* = 13)	*N* or mean (range)
Age (years)	12 (5–17)	Age (years)	40 (21–65)	Age (years)	9 (5–15)
		Age of child with life-limiting condition (years)	12 (0–17)	Age of child with life-limiting condition (years)	10 (3–16)
Gender	17:9	Gender		Gender	
Female:male		Female:male	30:10	Female:male	7:6
		Relationship to child		Relationship to child	
		Mother	29	Sister	7
		Father	10	Brother	6
		Sibling caregiver	1		
Diagnosis		Diagnosis of child		Diagnosis of child	
Cancer	6	Infectious disease	2	Metabolic	1
Metabolic	1	Cancer	6	Neurological	7
Neurological	5	Metabolic	9	Gastrointestinal	2
Respiratory	1	Neurological	10	Congenital	3
Gastrointestinal	10	Gastrointestinal	4		
Congenital	3	Genitourinary	1		
		Perinatal	1		
		Congenital	7		
Health and social care professionals (*n* = 15)		*N* or mean (range)	Commissioners (*n* = 12)		*N* or mean (range)
Gender			Gender		
* Female:Male*		14:1	* Female:Male*		11:1
Profession			Geographical location		
Palliative care nurse specialists		4	* Southeast England*		4
Children’s Community nurse		1	Greater London		1
* Hospice nurse*		1	East England		2
* Ward sister*		1	Northwest England		1
Paediatric palliative medicine consultant		1	Yorkshire and Humber		4
Haematology consultant		1			
General Paediatrician		1			
* Social worker*		1			
* Chaplain*		1			
* Psychologist*		1			
* Play specialist*		1			
* Physiotherapist*		1			

### Main findings

**Figure 1.**
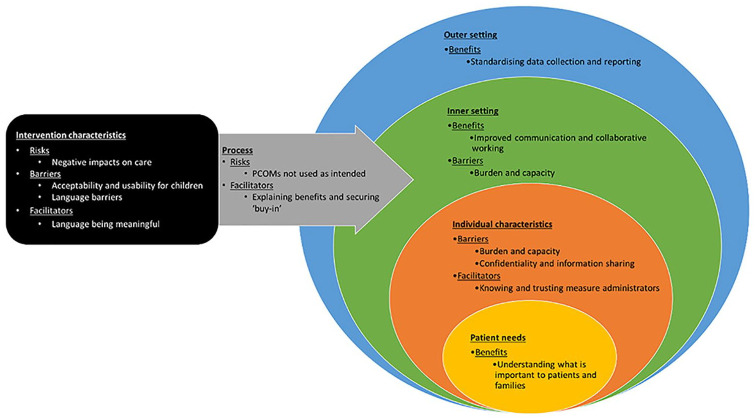
Themes and sub-themes mapped to adapted-CFIR domains.

Four main themes were constructed (anticipated benefits, risks, barriers and facilitators) with 13 sub-themes mapping across all 6 adapted-CFIR domains (see Figure 1). Utilising the adapted-CFIR in this way enabled better understanding of how factors might impact implementation across multiple levels. The themes and sub-themes are described in detail below.

### Theme 1: Anticipated benefits of using PCOMs

#### Understanding what is important to patients and families

All participants were supportive of the implementation of PCOMs into paediatric palliative care. One of the most frequently reported anticipated benefits for all stakeholder groups was the ability to enhance clinicians’ understanding about what is important to children and their families. Children actively wanted professionals to know and ask about the symptoms and concerns that matter to them

*‘If they knew what I thought was important, and what I liked, and who I was as a person, then I think that would help a lot more’*

Young Person aged 17 with cancer diagnosis

A further anticipated benefit was better identification of, and improved ability to meet, the needs of children and their families

*‘I think if you can have a tool that is universal [. . .] but is flexible enough that it really draws out what’s important for the child and family, I think we will be better as professionals at meeting those needs’*

Clinical Nurse Specialist

Although rarely discussed by parents in interviews, compared to children or professionals, one parent did recognise the benefit a PCOM could bring in terms of better understanding the needs of their children in the specific context of the family

*‘I think people have to understand what your priorities are to really be able to, as a family and in looking after your child, the whole picture to be able to meet and support you in the best way’*

Mother of 3-year-old with neurological diagnosis

#### Improved communication and collaborative working

One of the most commonly anticipated benefits among parent/carers and professionals was improvements to joined-up working and communication across teams and services

*‘everyone can be on the same page about what is important for the child and this family. So. . . the wider disciplinary team, because they might be under cardiology but actually they might need to keep in mind that actually this is what’s important, so they are not doing things that are unnecessary and they [the family] don’t want to happen’*

Trainee Clinical Nurse Specialist

Using PCOMs to share information with the wider care team about symptoms and concerns was seen as helpful by children and their families. Parents/carers felt it would reduce repetition in communication with different professionals

*‘I think erm. . .erm everybody that’s in his care [should have access], that have an involvement in his. . .because at least then they’re all seeing the same information and you’re not having to repeat yourself’*

Mother of 12 year old with congenital diagnosis

Standardising data collection and reporting

The benefit of standardised data collection and reporting was particularly important to commissioners. They discussed being able to use outcomes data to ensure that services they commissioned (and resource allocation) were best serving and meeting the needs of children and families

*‘one of the main incentives I can see is around whether we can secure additional funding and investment by demonstrating the outcomes because at the moment I think it feels like everybody who works in paediatric palliative care knows that it makes a really huge difference. And we agree but it’s really difficult to demonstrate’*

Commissioner

*‘any information that tells you about the needs of the services that you are commissioning is incredibly helpful. [. . .] I think it's all very useful as it helps you plan your services’*

Commissioner

### Theme 2: Anticipated risks in using PCOMs

#### Negative impacts on care

Whilst most participants felt that using PCOMs in routine care would have a positive impact on the care provided, two parent/carers raised concerns around the additional workload and the potential for this to negatively impact on provision of care

*‘Resources are always stretched and people are, you know the whole team are always busy, especially if they’re understaffed. And you don’t want to sort of create more work, more admin if that makes sense cos it’s going to affect their ability to care’*

Father of 1 year old with infectious disease

#### PCOMs not used as intended

Parents of children with neurological conditions raised concerns that completion of PCOMs could become a ‘tick box exercise’, which would just be another thing to do but with no real benefit to their child and family

*‘Yeah, it’s all. . .always to tick their boxes, that’s what it is. Like especially like with the social side of things, they have to tick’*

Mother of 15 year old with neurological condition

Similar concerns were echoed by a nurse regarding teenagers whether implementing a PCOM would truly enable active participation in their own care, and avoiding tokenistic inclusion

*‘like [teenagers] they’re actually taking part rather than they’re actually just being asked loads of questions’*

Nurse

### Theme 3: Potential barriers to implementing PCOMs

#### Acceptability and usability for children

Participants raised concerns around accessibility of a measure for children of different ages/cognitive abilities. This was emphasised as important to consider in the design of a measure to maximise acceptability and usability

*‘it will depend on the cognitive ability of the child [. . .] even if the child has a limited cognitive ability there are some basic questions you could ask them about, you know how. . .how they’re feeling and how. . .what their experiences have been. . .umm obviously you know. . .umm if they’ve got greater cognitive ability you can ask them more in-depth’*

Commissioner

*‘maybe not this one [points to example of an outcome measure] because what if you don’t know what mild pain is or the words’*

Child aged 15 with gastrointestinal diagnosis

#### Burden and capacity

Many professionals and commissioners noted that families were already being asked to complete several forms and expressed concern that they may not want to complete an additional measure. They also reported that children or their parents may not have the time or capacity, be that due to physical symptoms or emotional distress

*‘that maybe could be a little bit onerous sometimes when people have you know, perhaps pain or in emotional distress, they’re not really wanting particularly to fill in a bit of paper’*

Social worker and family therapist

*‘Cause they won’t want to fill something in that’s a million pages because they just don’t have the time’*

Trainee Clinical Nurse Specialist

Capacity was also raised as a concern primarily by commissioners with regards to the feasibility of implementing and using PCOMs in practice in relation to professionals’ workload

*‘it’s difficult because it takes time and space and effort and energy and in a system which is so stretched, actually that’s not always a priority’*

Commissioner

However, one commissioner noted that PCOMs were already used routinely in adult palliative care and therefore suggested it was possible that a PCOM could similarly fit into existing clinical workflows in the paediatric palliative care context

*‘I mean the community nurses could use it. Certainly, the adult one is used by district nurses. It’s used in inpatient hospice, it’s used [in] inpatient hospitals’*

Commissioner

#### Confidentiality and information sharing

Children (including siblings), primarily with cancer, congenital or gastrointestinal conditions, raised concerns regarding the sharing of information elicited through completion of a PCOM. Some were concerned about who the information might be shared with, often not wanting them to be shared beyond their parents or the professionals in the room. Some parent/carers and professionals also endorsed this view

*‘I just kind of want whoever asks me I just want to be like, just tell them or the people in the room. . . I don’t want it to go anywhere’*

Child aged 14 with Congenital Condition

*‘I think some people wouldn’t want everybody to know their business’*

Mother of 12 year old with cancer diagnosis

The importance of retaining control of the information, to protect family members, was also mentioned

*‘you know being asked taken aside and asked independently is quite nice, it just feels more personal, it doesn't worry the others as much’*

Sibling carer of 17 year old with cancer diagnosis

A similar potential barrier to using PCOMs was identified in relation to what parent/carers would allow professionals to share or discuss with the child

*‘We also need permission from parents on what to give them and that then has an impact on what we can hand out, depending ‘cause each child has very different information based on what the parents allow us to tell them’*

Nurse

*‘Some children [. . .] Their parents don’t even share what their care is or what’s happening’*

Mother 12 year old with cancer diagnosis

#### Language barriers

Language barriers when the child and family did not speak English fluently was a concern primarily raised by professionals. As translators were not always available, professionals found communicating and assessing children’s needs challenging

*‘So language is a really big problem. [. . .] when we’re having big discussions we can bring in our translators but we don’t have the ability to do that all the time. So that is difficult [. . .] we have had kids in the past where we have used faces and emotions where we have had more like what we are feeling to help them communicate as well. I think that’s really important especially for some of the overseas patients who are maybe like 6 or 7 but they don’t speak English. But it’s very, usually in a basic way’*

Ward Sister

One parent also discussed difficulties in communicating their child’s symptoms and concerns with professionals as English was not their first language

*‘English is not my first language, and I tell them what I can see but it’s so hard to explain’*

Mother of 1 year old with congenital condition

### Theme 4: Potential facilitators for implementing PCOMs

#### Explaining the benefits and securing ‘buy-in’

A key facilitator to successful implementation is educating or explaining potential benefits PCOMs may afford in practice. The importance of PCOMs being rigorously developed and evidence based was key to this explanation

*‘a lot of the time I think people in all kinds of organisations, but certainly in commissioning organisations, we have to explain that to people who don’t necessarily know as much about it as we do in a way that’s compelling erm and having a set of measures that are evidenced based and are used across more than just our services it would be really helpful to evidence that what we are doing is making a difference’*

Commissioner

The importance of explaining and demonstrating the benefits of PCOMs to teenagers in particular, to gain their buy-in, was echoed by children and professionals

*‘How it’s benefiting them. Especially the teenagers. If you know, you’re asking them and then they’re asking and being asked again and they go well what am I getting out of it?’*

Nurse

*‘sometimes there are days when I’m feeling like oh yes its fine, it’s all alright [to complete a questionnaire] and sometimes I’m feeling like, yeah it’s making me feel not okay, it’s making me feel uncomfortable [. . .] but at the same time it’s helpful and you know it’s for a good cause’*

Child aged 14 with cancer diagnosis

#### Knowing and trusting measure administrators

Drawing on existing rapport and trusted relationships was central to engaging children and families in using PCOMs. When children did not know or trust professionals, they felt less comfortable sharing their symptoms and concerns

*‘I think it would depend on who you sort of had that rapport with. Yeah, I don’t know. I mean for us it would probably be the nurses on the ward cos they just know us better’*

Mother of 12 year old with cancer diagnosis

*‘the nurses often don’t really know me and that’s why I’m often a bit like. . . weird around them’*

Child aged 12 with cancer diagnosis

Some children however, were less concerned about knowing and trusting the person administering the PCOM, and instead felt that it was more important that the person cared and would be able to help them

*‘I would, just someone who just has like compassion. Like erm. . .someone who’s not there just to get info, but someone who’s there actually to help the child and that’s. . .that’s what matters’*

Child aged 17 with gastrointestinal diagnosis

#### Language being meaningful

The language used in PCOMs was highlighted as an important design consideration to ensure acceptability and usability. Professionals suggested that using language that is meaningful, or mirrors words used by the child and family themselves, may reduce barriers to use

*‘I think it needs to be. . .I think it needs to be written in a language that is meaningful to families, more than to us. I think we can. . .it’s got to be easily accessible’*

Clinical Nurse Specialist

## Discussion

### Main findings

This study provides novel evidence on anticipated risks and benefits of, and barriers and facilitators to, the implementation of PCOMs into the care of children with life-limiting conditions and their families. Stakeholders recognised and welcomed the ability of PCOMs to improve understanding of child and family priorities, improve collaborative working and assist the standardisation of assessment outcome reporting.^6^ The greatest anticipated value of implementing PCOMS was in helping professionals better understand what was important to children and families, and helping commissioners ensure that services were meeting their needs.^46,47^

Feasibility of implementing and using PCOMs in routine practice is important to consider, particularly given concerns that additional workload could compromise care. Reported acceptability and implementability the C-POS: African Version suggests that a collaboratively-developed implementation strategy could facilitate the successful implementation of a similar measure into care for children with life-limiting conditions in the UK.^40^ PCOMs are used routinely across adult palliative care settings (including inpatient, acute and community/home settings) suggesting that a PCOM may be similarly incorporated into paediatric settings without negatively impacting clinical workflows or compromising care.^74,75^ Nevertheless, it is important to ensure that PCOMs are used in a meaningful way whereby PCOMs actively support children to participate in their own care and shared decision-making and to avoid it becoming tokenistic whereby children are just asked to complete a PCOM but with no additional discission, involvement or feedback.^6^ Given the expressed preference by children for measure completion to be integrated within conversations with their healthcare team,^49^ there may be impacts to clinical workflow beyond those identified in the adult care situation.

Barriers relating to measure design, staff time, skills and gatekeeping were anticipated; some of which can be overcome by robust design and psychometric testing.^49^ Concerns about respondent burden highlight the importance of designing measures that are quick to complete, to increase acceptability for patients and their families.^48,49^ Concerns relating to privacy and information sharing may be barriers to implementation and are often found to be linked to family members’ desire to protect each other.^23,76^ Shared-decision-making with parents about how and with whom PCOMs are completed^77^ along with more open communication about use and benefit of measures, may mitigate this potential barrier.

Understanding family dynamics was also important in terms of how and with whom measures should be completed. This must be managed sensitively by professionals for successful PCOM implementation in the paediatric setting. Children often want someone to talk to about their problems when completing a measure^49^ so measures may need to be completed in dialogue, and with a professional who has a relationship with the child and family. Potential barriers related to parents withholding information to protect their children,^78^ is a unique finding in relation to barriers that have been identified previously in adult palliative care settings^79^ or across paediatric settings more broadly,^62^ and similarly calls for consideration in how PCOMs are administered. Whether children and their families complete it together or separately must be considered as it may impact on the mode of administration and thus implementation and use of the measure in practice.^6^

As identified in previous work^80,81^ and noted in this study, for many children with life-limiting conditions and their families the lingua franca is not necessarily their primary language. Therefore, once developed and validated, it is also important that PCOMs are translated. This will ensure that PCOMs can support all children with life-limiting conditions and their families, irrespective of primary language or language proficiency and not introduce inequities in care, and potential disparities in outcomes.

Finally, recognition of the benefits of using PCOMs in routine paediatric palliative care is a particularly important facilitator for implementation. Research in adult palliative care^79^ and non-palliative paediatric settings^62^ suggests that recognition of potential benefits of PCOMs in practice often facilitates implementation through addressing barriers across multiple adapted-CFIR domains. Thus, the potential benefits of PCOMs should be emphasised with all stakeholders as part of implementation to encourage uptake and to ensure the benefits are realised for this population.

### What this study adds?

Whilst several barriers and facilitators for implementing PCOMs into paediatric palliative care have been identified in other paediatric settings,^62^ this work highlights barriers and facilitators that are specific to paediatric palliative care. We make eight recommendations for implementation and sustained use of new PCOMs into paediatric palliative care; displayed in Table 2 alongside how they have been informed by the findings of this study.

**Table 2.** Recommendations for implementation and sustained use of new person-centred outcome measures into care for children and young people with life-limiting and life-threatening conditions and their families.

Recommendation	Sub-themes informing recommendations
1. Children and families must be involved in the development of PCOMs to ensure that measure characteristics do not act as barriers to implementation	• Acceptability and usability for children • Language being meaningful
2. Strategies for implementation should be designed collaboratively with professionals to ensure they are optimal	• Negative impacts on care • PCOMs not used as intended • Burden and capacity
3. The benefits of PCOMs for care should be explained to families and professionals to facilitate implementation	• Explaining the benefits and securing ‘buy-in’ • Understanding what is important to patients and families • Improved communication and collaborative working • Standardising data collection and reporting
4. The professional administering the measure should be known and trusted by the child and family	• Knowing and trusting measure administrators
5. Completion of the measure should be a collaborative dialogue, in which the child, family and healthcare professional are fully involved where possible and appropriate	• Understanding what is important to patients and families • PCOMs not used as intended
6. Professionals should respond appropriately to issues raised through completion of the measure and ensure children and families are involved and informed of any changes in care as a result	• Understanding what is important to patients and families • PCOMs not used as intended
7. Discussions should be held with children and families to address privacy concerns, find out who they are comfortable with their information being shared with and to explain use and benefits of information-sharing in relation to PCOMs	• Confidentiality and information sharing • Explaining the benefits and securing ‘buy-in’ • Knowing and trusting measure administrators
8. Once robustly and scientifically developed and validated, PCOMs should be translated to locally relevant languages, to increase usability for families where the local language is not their primary language	• Language barriers • Language being meaningful

### Strengths and weaknesses

Using framework analysis facilitated the structured management of a large dataset, and the matrix function allowed for comparison between groups.^68^ This enabled the identification of factors that were specific to or common across the different participant groups, diagnoses or other participant or contextual factors. The use of the adapted-CFIR in the interpretation stage will enable the development of a theoretically informed strategy to support implementation of PCOMs in paediatric palliative care practice, shaped by key stakeholder perspectives.^10,11,82,83^

Due to the lack of existing measures in practice,^35^ the implementation of PCOMs was presented in a hypothetical way to interview participants. The original interview topic guides also did not include prompts related to specific implementation factors as described by the adapted-CFIR.^73^ Thus, further research is needed to understand what barriers may occur during implementation in practice in order to develop further strategies to facilitate implementation. Furthermore, this study was conducted in the UK through a western cultural lens and therefore the findings may not be as generalisable to other contexts.

## Conclusion

Understanding the perspectives of the different stakeholder groups within the paediatric palliative care setting has supported the identification of several context specific strategies and development of eight recommendations to support implementation of new PCOMs. Next steps will include the development of an implementation strategy specific to the UK paediatric palliative care context, and exploratory work to further understand how implementing a novel outcome measure would work in practice.

## Supplemental Material

sj-pdf-1-pmj-10.1177_02692163241234797 – Supplemental material for What are the anticipated benefits, risks, barriers and facilitators to implementing person-centred outcome measures into routine care for children and young people with life-limiting and life-threatening conditions? A qualitative interview study with key stakeholders Supplemental material, sj-pdf-1-pmj-10.1177_02692163241234797 for What are the anticipated benefits, risks, barriers and facilitators to implementing person-centred outcome measures into routine care for children and young people with life-limiting and life-threatening conditions? A qualitative interview study with key stakeholders by Hannah May Scott, Lucy Coombes, Debbie Braybrook, Daney Harðardóttir, Anna Roach, Katherine Bristowe, Myra Bluebond-Langner, Lorna K Fraser, Julia Downing, Bobbie Farsides, Fliss EM Murtagh, Clare Ellis-Smith and Richard Harding in Palliative Medicine
