# Supplementary material for: What are the anticipated benefits, risks, barriers and facilitators to implementing person-centred outcome measures into routine care for children and young people with life-limiting and life-threatening conditions? A qualitative interview study with key stakeholders
Source: Palliat Med. 2024 Mar 13;38(4):471–84. doi: 10.1177/02692163241234797 (PMC11025304; doi:10.1177/02692163241234797)
Supplement: sj-pdf-1-pmj-10.1177_02692163241234797 – Supplemental material for What are the anticipated benefits, risks, barriers and facilitators to implementing person-centred outcome measures into routine care for children and young people with life-limiting and life-threatening conditions? A qualitative int [file sj-pdf-1-pmj-10.1177_02692163241234797.pdf]

Topic Guides

For child participants, different topic guides were developed according to the following age groups: 5-7 years, 8-10 years, 11-15 years, 16-18 years. Due to the heterogeneity of this population, chronological age may not always reflect developmental age and therefore parent/caregivers were asked to advise which version was most appropriate for the child being interviewed. Topic guides for siblings were developed according to the following ages: 5-15 years and 16-18 years. Separate topic guides were developed for parent/carers of children over five years old and for parents of children under 5 years old. Separate topic guides were developed for professional and commissioner participants.

PCOMs were explained to participants as simple tools or measures that routinely ask children and their families about the symptoms and concerns that are important to them. The intention of PCOMS is to make sure children and families are asked about the things most important to them, help care teams know where to focus their efforts, and enable measurement over time. For professional and commissioner participants it was additionally noted that these measures are commonly used in the NHS and are usually called "person-centred outcome measures" or "patient-reported outcome measures".

Topic guides included questions around whether children and families were already routinely asked about the symptoms and concerns that were important to them, the acceptability and perceived benefit of asking these questions, potential challenges with using an outcome measure as described in care, who they would prefer to ask the questions, and have the answers shared with, and some additional design questions around recall, response format, administration mode.

Child aged 5-7

Of all the things you've just told me, what do you think matters most to you? What would you like doctors and nurses to do for you to help you?

Does anybody ever ask you about that? Would you like them to? Would it be OK if a doctor or nurse asked you about these things?

Who would you like to ask you (prompt staff, family others)?

If someone was feeling poorly how would you tell them? What do you say to tell a grown up so that you know that you are feeling poorly?

Child aged 8-10

Does anybody ever ask you about what matters most to you? Would you like them to?

Do you think it would help if your care team (doctors, nurses and others that help look after you) asked you about some of the things you have told me about? Would it be OK if they asked you each time they saw you? Or how often? And if they asked you about a problem, how far back do you think it would be best to remember, so perhaps how has this bothered you in the last day, 3 days, week- what is easiest to remember?

Who would you like to ask you (prompt staff, family others)?

And who could have that information you share about the things that bother you and matter to you- just your care team, you, your family? Why?

I'd really like to hear your ideas about how we could best ask what matters to you, if we were trying to find out how much of a problem something was for you from maybe not at all to bothering you very much, how could we ask that? Some ideas might be pictures showing things getting better or worse, or numbers from very low to high, you could mark it with a pen, you could do it on an ipad screen- what do you think you'd like to do?

The last thing I'd like to ask is what you think would be a good way to ask someone in a sentence if they are feeling better or worse than the last time they saw their team- what could we ask?

Child aged 11-15

Does anybody ever ask you about the things that are important to you? Would you like them to?

Do you think it would help if your care team (doctors, nurses and others that help look after you) asked you about some of the things you have told me about? Would it be OK if they asked you each time they saw you? Or how often? And if they asked you about a problem, how far back do you think it would be best to remember, so perhaps how has this bothered you in the last day, 3 days, week- what is easiest to remember?

Who would you like to ask you (prompt staff, family others)?

And who could have that information you share about the things that bother you and matter to you- just your care team, you, your family? Why?

I'd really like to hear your ideas about how we could best measure what matters to you, if we were trying to find out how much of a problem something was for you from maybe not at all to bothering you very much, how could we ask that? Some ideas might be pictures showing things getting better or worse, or numbers from very low to high, you could mark it with a pen, you could do it on an ipad screen- what do you think you'd like to do?

The last thing I'd like to ask is what you think would be a good way to ask someone in a sentence if they are feeling better or worse than the last time they saw their team- what could we ask?

Is there anything else you'd like to ask me or tell me?

Child aged 16-18

Does anybody ever ask you about the things that are important to you? Would you like them to?

Do you think it would help if your care team (doctors, nurses and others that help look after you) asked you about some of the things you have told me about? Would it be OK if they asked you each time they saw you? Or how often? And if they asked you about a problem, how far back do you think it would be best to remember, so perhaps how has this bothered you in the last day, 3 days, week- what is easiest to remember?

Who would you like to ask you (prompt staff, family others)?

And who could have that information you share about the things that bother you and matter to you- just your care team, you, your family? Why?

I'd really like to hear your ideas about how we could best measure what matters to you, if we were trying to find out how much of a problem something was for you from maybe not at all to bothering you very much, how could we ask that? Some ideas might be pictures showing things getting better or worse, or numbers from very low to high, you could mark it with a pen, you could do it on an ipad screen- what do you think you'd like to do?

The last thing I'd like to ask is what you think would be a good way to ask someone in a sentence if they are feeling better or worse than the last time they saw their team- what could we ask?

Is there anything else you'd like to ask me or tell me?

Parent/Caregiver

Do your care team routinely ask about these problems? Do you tell them? Does your child tell them? Why?

Do you tell your care team? DO they ask you? Why?

We are thinking about how to develop a simple measure that routinely asks you and your child about the main symptoms and concerns that affect people in your situation. The idea is that is makes sure you get asked about the important things, helps your care team to know where to focus their efforts, and allows us to measure how things are changing over time.

Do you think that would be useful?

How would it be best to complete this- child alone, you and child together, just you?

Which professionals do you think you'd like to complete it with? Who should have access to the information- just the care team- you, your child?

What do you think would be useful? Any challenges?

How could it be made easiest for your child to participate in the completion of the tool?

How could we best measure change- pictures, scores, iPad- what do you think would be easiest and most appealing?

If we were to ask each time if they were better or worse since last time, what words could we use?

Is there anything else you would like to tell us?

Parent/Caregiver proxy for children under 5

Do your care team routinely ask about these problems? How do they ask and who tells them?

As your child is so young, how do you decide what their daily needs are – how do you decide that the things you describe are a problem for the child, can you talk me though how you do it? Is this the same or different from how other family members and staff do it? How is it different?

How do staff usually find out what matters to your child? How do you try and make sure they know what matters?

We are thinking about how to develop a simple measure that routinely asks you and your child about the main symptoms and concerns that affect people in your situation. The idea is that is makes sure you get asked about the important things, helps your care team to know where to focus their efforts, and allows us to measure how things are changing over time.

Do you think that would be useful?

How would it be best to complete this for such a young child?

Which professionals do you think you'd like to complete it with? Who should have access to the information- just the care team- you, your child?

What do you think would be useful? Any challenges?

How could it be made easiest for your child to participate in the completion of the tool?

How could we best measure change- pictures, scores, iPad- what do you think would be easiest and most appealing?

If we were to ask each time if they were better or worse since last time, what words could we use?

Is there anything else you would like to tell us?

Siblings aged 5-15

We are trying to develop a simple tool that lists the things that matter to children facing illness and their family- what should it ask about (prompt physical, psychological social spiritual) prompt family parents/siblings?

Would you be happy to be asked?

Who should ask you?

Who should have that information apart from the care team? (PROMPT parents/child)

I'd really like to hear your ideas about how we could best measure what matters to you,

if we were trying to find out how much of a problem something was for you from maybe not at all to bothering you very much, how could we ask that? -Some ideas might be pictures showing things getting better or worse, or numbers from very low to high?

you could mark it with a pen, you could do it on an ipad screen- what do you think you'd like to do?

What would be the best time period to ask you about how you have been- a day? Three days? Week? What do you think?

The last thing I'd like to ask is what you think would be a good way to ask someone in a sentence if they are feeling better or worse than the last time they saw their team- what could we ask?

Is there anything else you'd like to ask me or tell me?

Siblings aged 16-18

What are the main things that you'd like support with? Would you be happy if the care team asked you about those things? Why?

We are trying to develop a simple tool that lists the things that matter to children facing illness and their family- what should it ask about (prompt physical, psychological social spiritual) prompt family parents/siblings?

Would you be happy to be asked?

Who should ask you?

Who should have that information apart from the care team? (PROMPT parents/child)

I'd really like to hear your ideas about how we could best measure what matters to you, if we were trying to find out how much of a problem something was for you from maybe not at all to bothering you very much, how could we ask that? Some ideas might be pictures showing things getting better or worse, or numbers from very low to high, you could mark it with a pen, you could do it on an ipad screen- what do you think you'd like to do?

What would be the best time period to ask you about how you have been- a day? Three days? Week? What do you think?

The last thing I'd like to ask is what you think would be a good way to ask someone in a sentence if they are feeling better or worse than the last time they saw their team- what could we ask?

Is there anything else you'd like to ask me or tell me?

Health and social care professionals

Do your care team routinely ask about these problems? Do the children and families volunteer these areas or do you have to ask?

What about how you do assessment- what are the communication challenges? How do you get round them?

We are thinking about how to develop a simple measure that routinely asks you and your child about the main symptoms and concerns that affect people in your situation. These are commonly used in the NHS and are usually called “person-centred outcome measures”. The idea is that you are able to ask about the important things, focus your time on their main symptoms and concerns, and see how things are responding over time.

Do you think that would be useful?

How would it be best to complete this- child alone, you and child together, you parent/carer and child?

Who should have access to the information- just the care team- you, your child?

What do you think would be useful? Any challenges? How would you use the information? What would make this as helpful as possible?

How could it be made easiest for children and families to participate in the completion of the tool?

How could we best measure change- pictures, scores, iPad- what do you think would be easiest and most appealing?

What would be a helpful recall period for the child?

If we were to ask each time if they were better or worse since last time, what words could we use?

Is there anything else you would like to tell us?

Commissioners

I'll ask you to tell me about how we could best incorporate outcome measures into routine care and reporting to commissioners. To start can you please tell me a little about your commissioning role, what do you see as the key role tasks and expectations?

PROM SECTION:

- It would help me to understand a little more if you could talk through the expectations of the patient-reported outcomes of the paediatric palliative care you commission?
    - What do you expect it to achieve?
- When a person attends, what do you expect is discussed with them?
- What are the priority patient-reported outcomes for you as a commissioner in routine care?
    - *prompting physical, psychological, social and spiritual areas*
- Do you currently have access to patient-reported outcome data on these priorities?
- Are there any outcomes you think are less important as a commissioner?
    - *prompting physical, psychological, social and spiritual areas*
- What currently do you think are the incentives to improve patient-reported outcomes from paediatric palliative care?
    - How might these change in the future?

We are thinking about developing a list of problems and concerns that matter most to people in relation to their paediatric palliative care so that they can "score" how they are on each of the key items. This approach i.e. Patient Reported Outcome Measures or PROMS is commonly used in the NHS for a wide range of health conditions. The idea is that it enables the individual to identify their problems and priorities, it enables health care providers to focus on what really matters to the person receiving care, and is a way of monitoring over time whether the care delivered is improving health.

- We are trying to ensure that the tool reflects what matters to people in the UK, and also to ask how it should be used so that it is acceptable and useful. What do you think of the idea?
    - Probe why?
- Earlier on in the interview, I asked you about the priority areas for paediatric palliative care. Given that we plan to develop this tool or short list, as a commissioner what would you DEFINITELY want to see included in that list?
- Thinking about the format for this tool, roughly how many questions would you like it to include?
- What would be useful for you as commissioners from information on patient-reported outcomes?
- Are there any outcomes that you think are not relevant for you as a commissioner?
- How could a PROM support your work as a paediatric palliative care commissioner?
- How would you like the information to be shared?
- How could the information be used to improve care?

- What time period do you think people can reliably recall (e.g. “in the past X days I have been having problems with…..”)

That is the end of my questions, is there anything else you’d like to share with me?
